# Whole-Genome Sequence of Pseudomonas putida Strain 1312, a Potential Biostimulant Developed for Agriculture

**DOI:** 10.1128/MRA.01073-18

**Published:** 2018-09-13

**Authors:** Julien Crovadore, Damien Grizard, Bastien Cochard, Romain Chablais, Philippe Blanc, François Lefort

**Affiliations:** a Plants and Pathogens Group, Research Institute Land Nature and Environment, hepia, HES-SO University of Applied Sciences and Arts Western Switzerland, Jussy, Geneva, Switzerland; b Proxis Développement, Levallois-Perret, France; c Bioprox, Noyant, France; University of Arizona

## Abstract

We report the draft genome sequence of strain 1312 of Pseudomonas putida, which could be interesting to develop as a biostimulant for agriculture and soil depollution treatments.

## ANNOUNCEMENT

Pseudomonas putida (Trevisan 1889) Migula 1895 is a Gram-negative saprotrophic and innocuous rod-shaped bacterium and a ubiquitous resident of soils, water, and plant rhizospheres. It grows optimally in the range of 25 to 30°C (1–3). Many works reported stimulating effects on crop yield and health due to auxin synthesis, siderophores, and antibiotic production and the elicitation of induced systemic resistance against pathogenic bacteria and fungi (4–9). Some strains may cause nosocomial infections in immunodepressed patients (10, 11). P. putida is also known for degrading aliphatic and aromatic compounds and can be used for soil bioremediation (12, 13).

The strain 1312 was isolated from soil in France and identified as P. putida by metabolic profiling. DNA was extracted with a modified cetyl trimethylammonium bromide (CTAB) protocol (14) from a culture grown from a single colony to its exponential phase in King’s B broth. The library was prepared using the TruSeq Nano DNA library preparation kit (Illumina, USA).

Whole-genome sequencing was carried out in one Illumina MiniSeq run at 2 × 151-bp paired-end read length using a MiniSeq high-output kit, which provided 11,200,000 reads and a genome coverage of 232×. Overall quality metrics of the reads (Phred quality score per base, per sequence, etc.) were assessed with FastQC version 0.11.5 (http://www.bioinformatics.babraham.ac.uk/projects/fastqc). The genome assembly, computed with the SPAdes genome assembler version 3.10 (15) and set in “paired end assembly, careful mode,” yielded 91 contigs (≥200 bp), which were arranged with BioEdit version 7.0.5 (16) and analyzed with QUAST version 4.6.3 (17), set at “QUAST skip contigs shorter than 200 bp.” The genome total length was 6,766,660 bp, with a GC content of 62.06% and an *N*_50_ value of 146,305 bp. Its complete 16S rRNA gene sequence showed 99.9% identity with more than 20 P. putida strains in the GenBank 16S database.

A public gene annotation was provided by the NCBI Prokaryotic Genome Automatic Annotation Pipeline (PGAP) version 4.1 (18). Annotation was also done with the Rapid Annotations using Subsystems Technology (RAST) server version 2.0 (19) with the ClassicRAST annotation scheme. PlasmidFinder version 1.3 (20) and plasmidSPAdes (21), both with default settings, did not detect any plasmids. The RAST server identified 5,993 coding sequences and 80 RNA genes, and PGAP retrieved 6,060 genes and 85 RNA sequences. No known toxin or virulence genes were detected. Pathways involved in nitrogen metabolism counted 2 nitrilase, 5 nitrate reductase, and 3 nitrile reductase genes. Five genes were involved in auxin synthesis. Forty-six genes provide siderophore sensing, transport, and reception, and 26 genes participate in pyoverdine synthesis. This strain harbors 56 genes for 2 complete type II and VI secretion systems. The type VI secretion system (T6SS) is used by P. putida to deliver antibiotics in the environment for targeting numerous bacterial species (22). The presence of 218 genes involved in the metabolism of aromatic compounds suggests that this strain could degrade a variety of such compounds. With the identification of 130 genes, we can predict resistance to antibiotics and metals. The presence of 45 genes involved in motility suggests that this strain should be motile. This strain has shown a biostimulant effect on small-scale experiments in a climatic chamber (our unpublished data).

### Data availability.

This whole-genome shotgun project was deposited at DDBJ/EMBL/GenBank under the accession number NFSB00000000. The version described in this paper is the first version, NFSB01000000. The 91 contigs have been deposited under the accession numbers NFSB01000001 to NFSB01000091. Raw sequencing data sets have been registered in the NCBI Sequence Read Archive database (23) under the accession number SRR5513018.
